# Immunological observations and transcriptomic analysis of trimester‐specific full‐term placentas from three Zika virus‐infected women

**DOI:** 10.1002/cti2.1082

**Published:** 2019-11-05

**Authors:** Fok‐Moon Lum, Vipin Narang, Susan Hue, Jie Chen, Naomi McGovern, Ravisankar Rajarethinam, Jeslin JL Tan, Siti Naqiah Amrun, Yi‐Hao Chan, Cheryl YP Lee, Tze‐Kwang Chua, Wearn‐Xin Yee, Nicholas KW Yeo, Thiam‐Chye Tan, Xuan Liu, Sam Haldenby, Yee‐sin Leo, Florent Ginhoux, Jerry KY Chan, Julian Hiscox, Chia‐Yin Chong, Lisa FP Ng

**Affiliations:** ^1^ Singapore Immunology Network Agency for Science, Technology and Research Singapore Singapore; ^2^ Institute of Molecular and Cell Biology Agency for Science, Technology and Research Singapore Singapore; ^3^ Department of Pathology National University Health System Singapore Singapore; ^4^ KK Women's and Children's Hospital Singapore Singapore; ^5^ Department of Pathology University of Cambridge Cambridge UK; ^6^ NUS Graduate School for Integrative Sciences and Engineering National University of Singapore Singapore Singapore; ^7^ Department of O&G KK Women's and Children's Hospital Singapore Singapore; ^8^ Centre for Genomic Research Institute of Integrative Biology University of Liverpool Liverpool UK; ^9^ NIHR Health Protection Research Unit in Emerging and Zoonotic Infections University of Liverpool Liverpool UK; ^10^ Communicable Diseases Centre Institute of Infectious Diseases and Epidemiology Tan Tock Seng Hospital Singapore Singapore; ^11^ National Centre for Infectious Diseases Singapore Singapore; ^12^ Saw Swee Hock School of Public Health National University of Singapore Singapore Singapore; ^13^ Lee Kong Chian School of Medicine Nanyang Technological University Singapore Singapore; ^14^ Department of Reproductive Medicine KK Women's and Children's Hospital Singapore Singapore; ^15^ KK Research Centre KK Women's and Children's Hospital Singapore Singapore; ^16^ Cancer and Stem Cell Program Duke–NUS Medical School Singapore Singapore; ^17^ Institute of Infection and Global Health University of Liverpool Liverpool UK; ^18^ Department of Paediatrics KK Women's and Children's Hospital Singapore Singapore; ^19^ Lee Kong Chian School of Medicine Nanyang Technological University Singapore Singapore; ^20^ Yong Loo Lin School of Medicine National University of Singapore Singapore Singapore; ^21^ Graduate Medical School Duke‐NUS Medical School Singapore Singapore; ^22^ Department of Biochemistry Yong Loo Lin School of Medicine National University of Singapore Singapore Singapore

**Keywords:** histology, infection, placenta, RNA‐seq, transcriptomics, Zika virus

## Abstract

**Objectives:**

Effects of Zika virus (ZIKV) infection on placental development during pregnancy are unclear.

**Methods:**

Full‐term placentas from three women, each infected with ZIKV during specific pregnancy trimesters, were harvested for anatomic, immunologic and transcriptomic analysis.

**Results:**

In this study, each woman exhibited a unique immune response with raised IL‐1RA, IP‐10, EGF and RANTES expression and neutrophil numbers during the acute infection phase. Although ZIKV NS3 antigens co‐localised to placental Hofbauer cells, the placentas showed no anatomic defects. Transcriptomic analysis of samples from the placentas revealed that infection during trimester 1 caused a disparate cellular response centred on differential eIF2 signalling, mitochondrial dysfunction and oxidative phosphorylation. Despite these, the babies were delivered without any congenital anomalies.

**Conclusion:**

These findings should translate to improve clinical prenatal screening procedures for virus‐infected pregnant patients.

## Graphical abstract

Zika virus infection during pregnancy is associated with neurologic birth defects, but the effects on placental development are unclear. In this study, each woman exhibited a unique immune response, but they collectively diverged from healthy controls with raised IL‐1RA, IP‐10, EGF and RANTES expression and neutrophil numbers during the acute infection phase. Transcriptomic analysis of samples from the placentas revealed that infection during trimester 1 caused a disparate cellular response centred on differential eIF2 signalling, mitochondrial dysfunction and oxidative phosphorylation. These findings should translate to improve clinical prenatal screening procedures for virus‐infected pregnant patients.

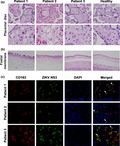

## Introduction

Zika virus (ZIKV) caused numerous outbreaks of infection worldwide,^1^ and the scale of these outbreaks highlighted several features of ZIKV infection that had previously been unrecognised or under‐reported. One such feature included congenital foetal growth‐associated anomalies as a result of ZIKV infection during pregnancy, termed congenital Zika syndrome (CZS).^2^, ^3^ Pregnancy is divided into three trimesters, based on the series of developmental changes that occur in the foetus and the physiological changes that occur in the mother. Understanding the nature of the ZIKV–host interactions at each trimester is required to determine the consequences of maternal–foetal ZIKV transmission. Important physiological changes occur in women during pregnancy, including immune responses.^4^ While the unique immunologic state experienced during pregnancy prevents the mother from rejecting the foetus,^5^ the state of pregnancy could increase the mother's susceptibility to infection.^6^

The placenta develops from trophectoderm surrounding the blastocyst and rapidly grows after implantation to establish the foetal life support system before the rapid growth of the foetus in the second half of pregnancy. The placenta is the physical barrier between the mother and her foetus. It is the site of transport of oxygen nutrients, antibodies and waste products between mother and baby.^7^ Importantly, the placenta also protects the foetus from infections, exhibiting a robust innate and adaptive immune response to pathogens.^8^ Hofbauer cells are macrophages located within the stroma of the placenta and can be isolated from the placenta and be infected by ZIKV experimentally.^9^ Positive ZIKV infection has been detected in placental tissue^10^ and foetuses^3^ of women who have terminated their pregnancies, suggesting the virus can evade placental immune defence mechanisms. As the risk of congenital anomalies, including microcephaly, is largely associated with ZIKV infection during the first trimester of pregnancy,^11^ it is also possible that ZIKV infection perturbs placental development. Such perturbations could also upset the placental immune function. The mechanisms underlying how ZIKV infection in the three trimesters affects the placental cellular responses, especially in pregnancies without any congenital complications, are currently unknown. Understanding these provides an alternate perspective regarding ZIKV pathogenesis in asymptomatic pregnant patients.

Here, the placental cellular responses in ZIKV‐infected pregnant women were probed and the study delineated how ZIKV infection affected placental development in each trimester. Three pregnant women were recruited to the study who had been infected with ZIKV during the first, second or third trimesters. After successful, full‐term delivery of healthy infants, the placentas were harvested and separated into the placental discs (chorionic villi) and foetal membranes (chorion and amnion) to investigate the cellular responses in these anatomically distinct structures. Histology and immunofluorescence analyses were used to identify any tissue parenchymal abnormalities and immune‐cell changes were determined by immunophenotyping. This was combined with RNA sequencing (RNA‐seq) to determine the transcriptomic profiles of these samples. Although the number of ZIKV‐infected pregnant women recruited was low and may not represent the entire population, it still provides insightful data on the possible placental responses during trimester‐specific ZIKV infection.

## Results

### Pregnant women in different trimesters exhibited differential susceptibility to ZIKV infection

Three ZIKV‐positive pregnant women were recruited to the study, who had been infected with ZIKV in different trimesters (Table 1) following the ZIKV outbreak that happened in Singapore in 2016.^12^ These patients suffered from mild symptoms such as fever, rash and headache. Only one patient experienced pain in her eyes. Blood samples were taken during the acute [5–6 days post‐illness onset (PIO)] and convalescent (9–14 days PIO) phases of infection, and analysed for the presence of ZIKV NS3 antigens.

**Table 1:** Profiles of ZIKV‐infected pregnant patients

	Acute phase			Convalescent phase			Healthy[Link]
	Patient 1	Patient 2	Patient 3	Patient 1	Patient 2	Patient 3	
Sample collection (PIO)[Link]	5	7	6	14	13	9	–
Age (years)	33	34	28	–	–	–	–
Presence of symptom[Link]							
Fever	No	No	Yes	–	–	–	–
Headache	Yes	No	Yes	–	–	–	–
Rash	Yes	No	No	–	–	–	–
Pain around eye area	Yes	No	No	–	–	–	–
Presence of ZIKV RNA in urine[Link]	Yes	Yes	Yes	–	–	–	–
Percentage ZIKV antigen in blood (infection level)[Link]	15 (moderate)	0.44 (low)	2.66 (low)	7.61 (low)	0.19 (low)	10.1 (moderate)	–
Cell counts (numbers μL^−1^ blood)[Link]							
Neutrophils	9378	4915	4868	8934	3668	5936	3784 ± 428
Monocytes	243	257	37	323	239	99	253 ± 96
CD4 T cells	301	954	393	501	491	407	993 ± 105
CD8 T cells	129	341	125	237	236	154	358 ± 83
DNT cells	41	30	27	132	29	25	93 ± 30
NK cells	57	72	126	232	103	115	163 ± 75
NKT cells	81	53	79	264	50	69	87 ± 39
CD56hi cells	16	13	15	28	12	15	11 ± 1
CD14^+^CD56^+^ cells	30	43	9	41	43	29	31 ± 13
B cells	74	309	85	105	114	82	211 ± 100
ZIKV‐specific antibody titre[Link]							
IgM	1.29	1.91	2.72	0.92	1.79	2.68	–
IgG	1.10	3.79	3.14	0.88	3.85	2.86	–

Average numbers of peripheral blood immune cells obtained from healthy control are shown as mean ± SD.

Samples were collected during both acute [1–7 days post‐illness onset (PIO)] and convalescent (8–14 days PIO) phases. Patients 1, 2 and 3 were infected during the first, second and third trimester, respectively.

Presence of any symptoms was recorded during the patients’ first visit to the hospital.

Presence of Zika virus (ZIKV) RNA in urine samples was determined by qRT‐PCR during the patients’ first visit to the hospital.

Infection is determined by the percentage of ZIKV Ag‐positive CD14^+^ monocytes, and the level of infection can be classified as moderate (>10%) or low (< 10%).

Cellular numbers of the peripheral blood immune subset were calculated with the following formula: (Percentages of specific immune subset × total leucocyte numbers = cellular numbers of specific immune subset). Percentages of specific immune subset were obtained with immune‐phenotyping. Total leucocyte numbers were obtained with a haematology analyser.

Antibody titre is expressed as fold change increase relative to levels obtained in healthy controls.

CD14^+^ monocytes are the predominant cellular target of ZIKV.^13^ Interestingly, each patient showed a different ZIKV NS3 antigen profile in CD14^+^ monocytes. Firstly, 15% of Patient 1's CD14^+^ monocytes were positive for ZIKV NS3 antigens, which persisted to a lesser degree in the convalescent phase (7.61%). Secondly, in Patient 2, ZIKV NS3 antigens were barely detectable in CD14^+^ monocytes at both time points (0.19–0.44%). Lastly, Patient 3 exhibited a ‘delayed’ infectious response, in which 2.66% of CD14^+^ monocytes were positive for ZIKV NS3 antigen levels during the acute phase, which increased to 10.1% of total CD14^+^ monocytes during the convalescent phase (Table 1 and Supplementary figure 1a, b). The different infection profiles exhibited by the three patients were corroborated by variations observed in their blood immune‐cell numbers (Table 1 and Supplementary figure 2a, b) and ZIKV‐specific antibody titres (Table 1 and Supplementary figure 1c, d). Interestingly, ZIKV‐specific antibody production was weak in Patient 1, perhaps indicating a direct consequence of ZIKV infection on the host humoral immune response during the first trimester. Titres of ZIKV‐specific antibodies (both IgM and IgG) did not change during the convalescent phase in both Patients 2 and 3 (Table 1 and Supplementary figure 1c, d).

### ZIKV antigen localised to placental Hofbauer cells and persisted until birth

**Figure 1:**
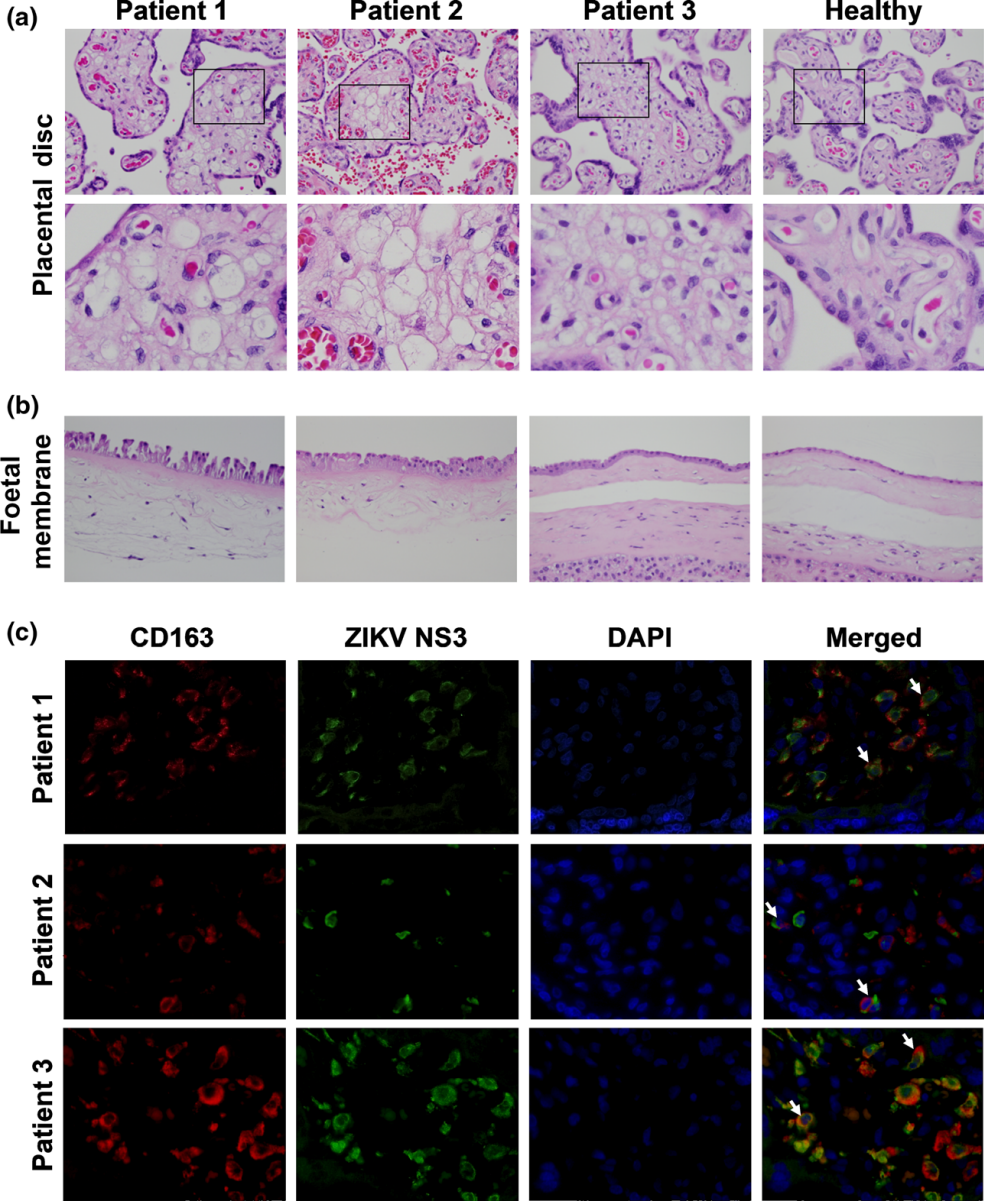
Zika virus (ZIKV) NS3 antigen in CD163‐positive macrophages impacts stromal changes in full‐term placentas. Representative haematoxylin and eosin (H&E)‐stained sections showing the placental disc (chorionic villi) and foetal membranes of three patients infected by ZIKV at different gestational ages. **(a)** Aggregates of vacuolated cells morphologically in keeping with Hofbauer cells are observed in the chorionic villi of all three infected patients. Areas highlighted by the black boxes are magnified in the lower panel. **(b)** Increased stromal and mononuclear cells are noted in the subamniotic connective tissue of the foetal membranes of the infected patients. All images were captured at 40× magnification. **(c)** Immunofluorescence microscopy was used to visualise ZIKV NS3 antigen (green) and CD163 protein (red) in the full‐term placentas of the ZIKV‐infected patients. The white arrows indicate co‐localisation of the ZIKV antigen and CD163 protein within the villous stroma of the placental disc. All images were captured at 40× magnification.

All three patients successfully carried infants to term with no microcephaly or CZS, and infants were delivered via normal vaginal deliveries with no intrapartum complications. Once delivered, the full‐term placenta was separated into placental discs and foetal membranes, and a series of histologic analyses were performed. By haematoxylin and eosin (H&E) staining of the placental discs, villous maturity within the normal limits of a term placenta was observed in all three cases. The chorionic villi of all three placental discs showed patchy dilatation featuring stromal oedema and prominent aggregates of vacuolated cells. These vacuolated stromal cells have the morphologic features of villous stromal macrophages, that is Hofbauer cells. No features of ongoing or remote acute or chronic villitis, intervillositis, villous necrosis or fibrosis were observed (Figure 1a). This indicated that ZIKV infection did not induce any overt adverse placenta pathology. The foetal membranes of all three placentas also showed several reactive changes, including increased numbers of vacuolated mononuclear cells within the subamniotic connective tissue, in keeping with increased infiltration of macrophages. In addition, the amniotic epithelial cells exhibited columnar cell metaplasia, being more pronounced in placentas infected in the first and second trimesters. There are no signs of acute chorioamnionitis (Figure 1b).

Immunofluorescence staining of the placental discs confirmed the presence of CD163^+^ Hofbauer cells, which exhibit functions similar to those of M2‐like macrophages.^14^ Counter‐staining with a ZIKV NS3‐specific antibody^13^ indicated that ZIKV protein co‐localised to Hofbauer cells (Figure 1c), in line with previous reports.^9^ Staining of placental discs with secondary antibody alone did not result in any signals (Supplementary figure 3). Thus, this ruled out autofluorescence and indicated positive infection of the placenta, regardless of the pregnancy trimester in which ZIKV infection occurred. Notably, these data showed that ZIKV proteins were present in the placenta up to delivery, without causing any physical harm to the newborn infant. However, it is important to continue monitoring the newborn as anomalies may manifest at a later time.^15^

### The ZIKV immune response correlated with the level of ZIKV infection in CD14^+^ monocytes

The immunologic response to ZIKV infection in the three pregnant women was compared between the acute and convalescent time points, which will provide important insights on how the host response is affected by the infection in different trimesters. Blood plasma was assayed for 42 different immune mediators, including pro‐inflammatory and anti‐inflammatory cytokines, chemokines and growth factors. Plasma samples from healthy, non‐pregnant females were used as controls. To segregate the patients based on the immunologic response, the derived data were processed by multiple factor analysis (MFA),^16^ which collectively analysed different measurements made from the same set of individuals. Both data sets were specified as two different groups of measurements to the algorithm.

**Figure 2:**
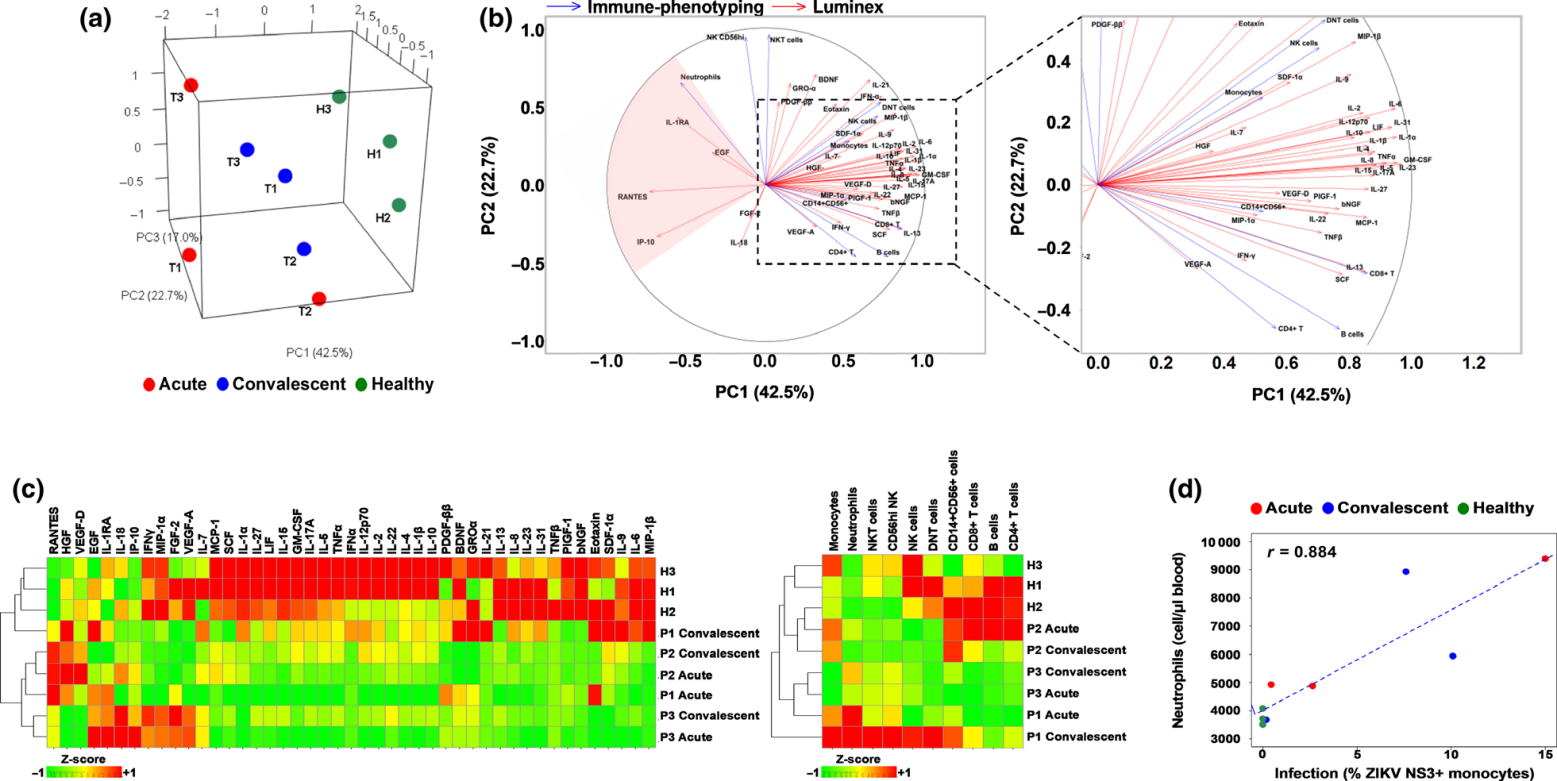
Immune characterisation of Zika virus (ZIKV)‐infected pregnant women. Blood samples were obtained from three pregnant women during the acute and convalescent phases of ZIKV infection. The women were infected in different trimesters of their pregnancy: P1, trimester 1; P2, trimester 2; and P3, trimester 3. Levels of peripheral blood immune cells and immune mediators were quantified by Luminex and immunophenotyping, respectively. Healthy controls (*n* = 3) were included. **(a)** Principal component analysis scores of the infected and healthy women based on a collective analysis of immunophenotyping and cytokine measurements with multiple factor analysis (MFA). **(b)** Correlation of immunophenotyping and cytokine measurements with the first two principal component (PC1 and PC2) axes of the MFA. **(c)** Heatmaps comparing the cytokine and immunophenotyping data for each subject. **(d)** Correlation between infection (% ZIKV NS3^+^ monocytes) and neutrophil numbers in whole blood samples.

The unsupervised data analysis clearly segregated the samples according to healthy controls and ZIKV‐infected patients and to convalescent and acute phases of infection along the first principal component (PC1) of the data. PC1 accounted for 42.5% of the variation in the measurements across the samples (Figure 2a). The second and third principal components (PC2 and PC3) together accounted for 39.7% of the variation between measurements and highlighted the differences between individuals. Along these axes, the patient samples from the convalescent phase of infection were closely grouped together compared to those from the acute phase of infection.

Correlation of measurements with PC1, which separated ZIKV‐infected patients and healthy controls, showed that a subset of immune mediators (IL‐1RA, EGF, RANTES and IP‐10) and neutrophils were associated with acute ZIKV infection (Figure 2b, c). Noteworthy, neutrophil numbers in the peripheral blood positively correlated with the percentage of ZIKV‐infected (ZIKV NS3^+^) monocytes (Figure 2d and Table 1).

### ZIKV infection during the first trimester of pregnancy triggered disparate eIF2 signalling and oxidative phosphorylation in the placenta

Transcriptomic profiling of the placental discs and foetal membranes of the placentas was used to investigate the cellular host response during ZIKV infection. Both tissues were digested to obtain a single‐cell suspension: the placental disc was further separated into CD45^+^ immune and CD45^−^ non‐immune fractions, to define the drivers of ZIKV‐induced cellular changes. Tissue samples were collected in triplicate to account for tissue heterogeneity, except those obtained from Patient 3, where the sample was processed as a whole, because of technical difficulties. Placental samples from two healthy women were included as controls.

**Figure 3:**
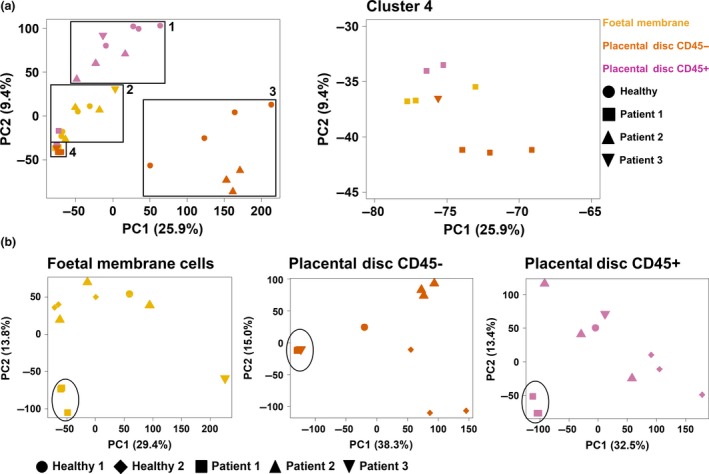
Principal component analysis of gene expression in placental samples. Full‐term placentas from Zika virus‐infected pregnant patients were obtained and separated into the foetal membrane, and CD45^−^ non‐immune and CD45^+^ immune cells of the placental disc. Placentas from two healthy women were obtained in parallel as a control. The transcriptomes of the three cell types (placental disc CD45^+^, placental disc maternal CD45^−^ and foetal membrane cells) were assessed by RNA sequencing (RNA‐seq). Principal component analysis was performed by analysing RNA‐seq data either **(a)** collectively from all three cell types or **(b)** individually as each specific cell type.

Over 517 million paired‐end RNA‐seq reads were obtained with a median of > 15 million paired‐end reads per sample mapped to the human transcriptome, and 29 613 genes had a detectable expression level of > 1 transcript per million (TPM) per sample. Principal component analysis (PCA) of the gene expression profiles segregated the samples by their origin (placental disc or foetal membrane), regardless of whether they were derived from patients or controls (Figure 3a). Patient 1 was the only exception, as the gene expression data all clustered together (Figure 3a, cluster 4). This effect was further elaborated when PCA was performed separately for each tissue type (Figure 3b), where samples from Patient 1 segregated from the rest.

To determine the cellular differences in the placenta of Patient 1, the unique differential gene expression (DGE) signature was compared to healthy controls. A large number of DEGs were identified between the placentas of Patient 1 and those from healthy controls (placental disc CD45^+^, *n* = 674; placental disc CD45^−^, *n* = 2780; and foetal membrane, *n* = 2467). Conversely, few to no DEGs were identified between the placentas derived from Patient 2 and healthy controls (placental disc CD45^+^, *n* = 0; placental disc CD45^−^, *n* = 51; and foetal membrane, *n* = 1; Supplementary figure 4a). Unfortunately, samples from Patient 3 could not be analysed because of an insufficient number of replicates.

**Figure 4:**
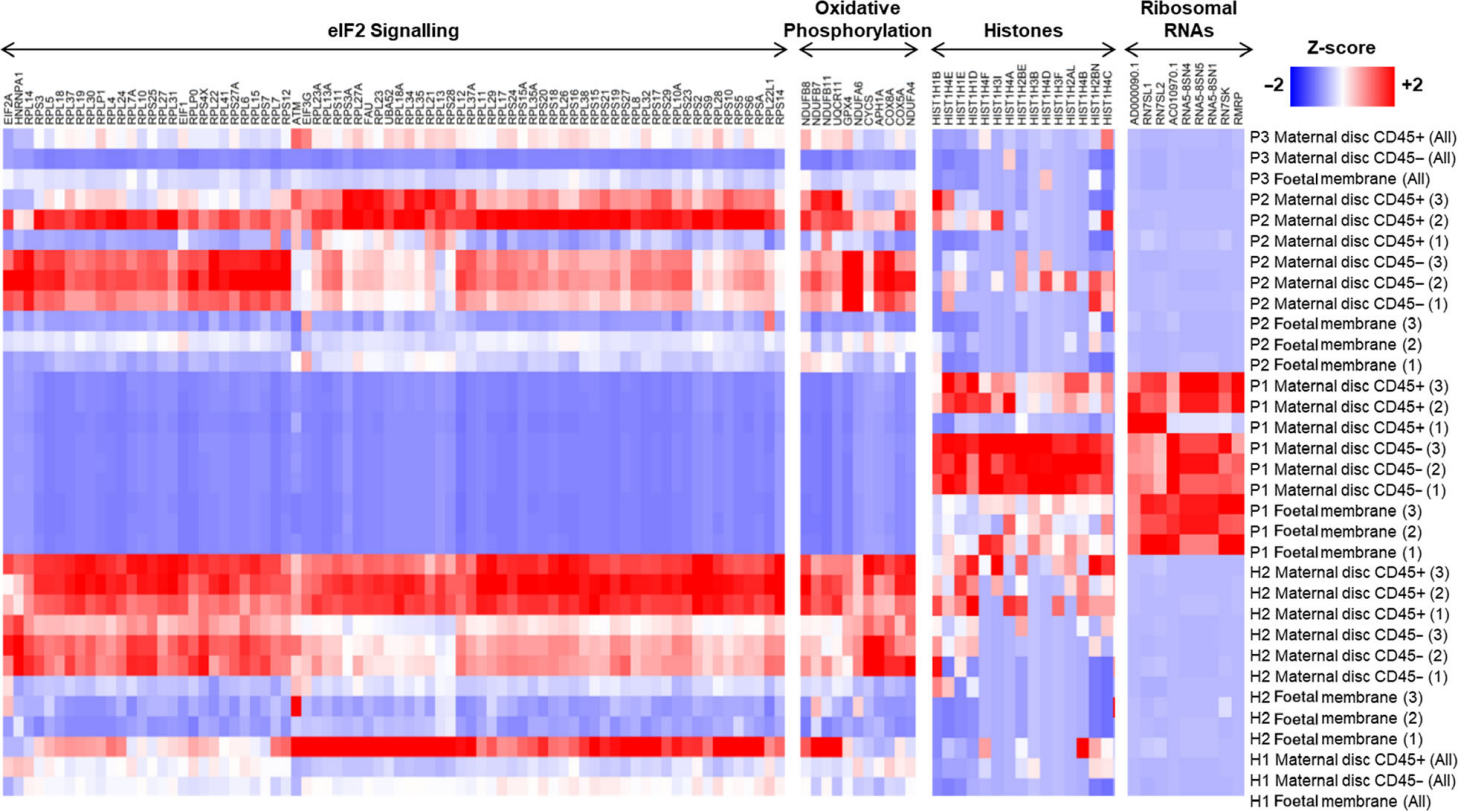
Placental gene expression associated with Zika virus (ZIKV) infection. Heatmaps comparing gene expression in the different placental specimens (placental disc CD45^+^, placental disc CD45^−^ and foetal membrane cells) from ZIKV‐infected women infected during the first (P1), second (P2) or third (P3) trimesters of pregnancy. Placental samples from two healthy women (H1 and H2) were obtained in parallel as a control. Samples from H2, P1 and P2 were processed in triplicates. The most highly differentially regulated genes and pathways in P1 included EIF2 signalling, oxidative phosphorylation, histones and ribosomal RNAs. Gene expression measurements from RNA‐seq in transcript per million (TPM) scale were used to calculate a Z‐score on each row.

The potential functions of the DEGs in placental tissue from Patient 1 were analysed using gene ontology (GO), pathway enrichment and gene set enrichment analysis (GSEA) to provide a global overview. In the CD45^−^ fraction of the placental disc from Patient 1, GO analysis identified several DEGs as ribonucleoproteins, mitochondrial genes and histones (Figure 4 and Supplementary table 1). Ingenuity Pathway Analysis identified the role of eIF2 signalling, as the main canonical pathway associated with these DEGs (Supplementary figure 4b). The eIF2 pathway regulates global protein synthesis and translation in response to stress. Oxidative phosphorylation was also significantly modulated, where 90% of genes involved in this pathway showed a reduced expression by > 2‐fold compared to healthy controls. An increased abundance of histone transcripts during viral infection has been previously reported^17^, ^18^ and was also observed in Patient 1 (Figure 4). The data also suggested potential modulation of oxidative phosphorylation and eIF2 signalling in both the CD45^+^ cells of the placental disc and foetal membrane in the placenta from Patient 1 (Figure 4, Supplementary figure 4b and Supplementary table 2).

**Figure 5:**
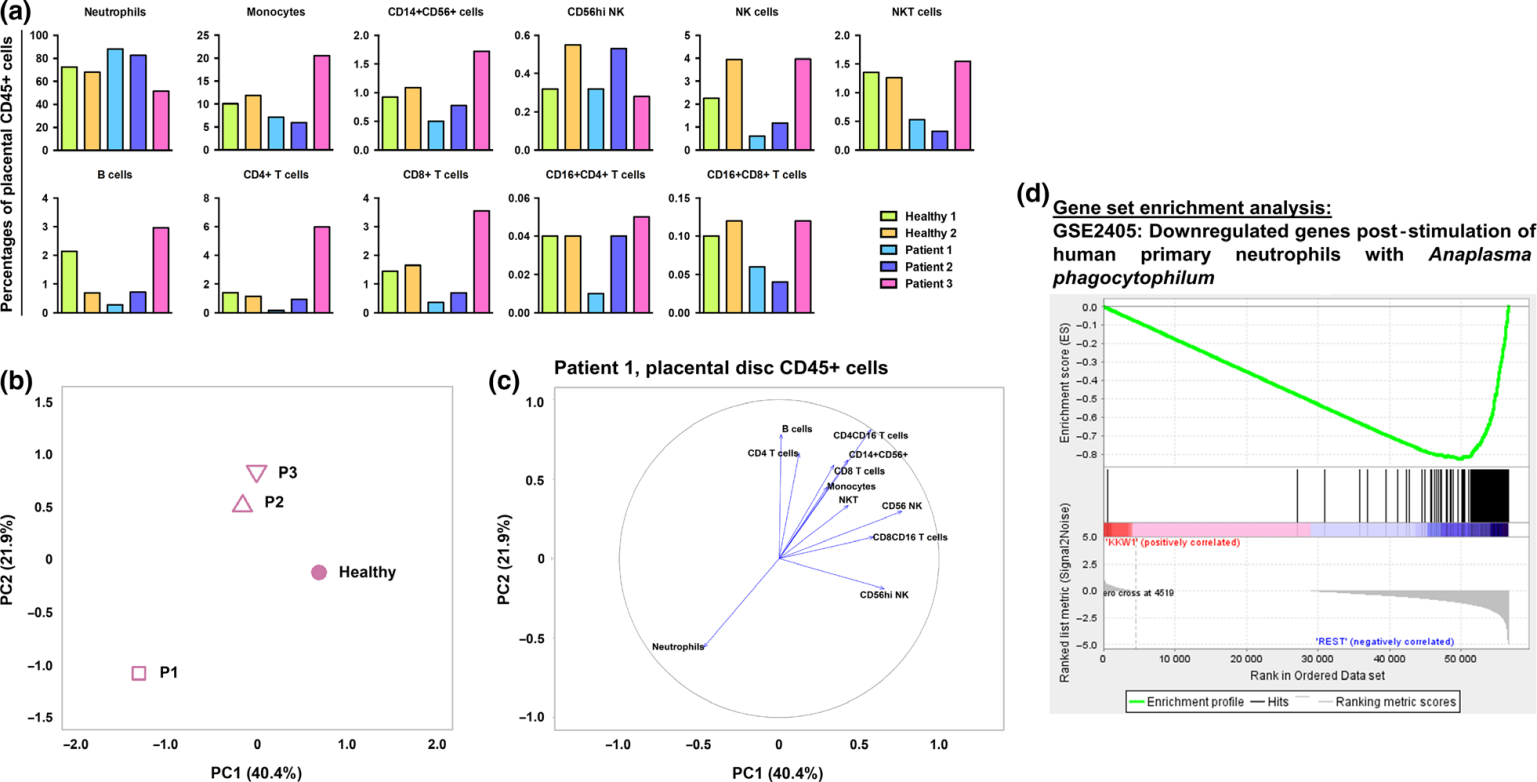
Zika virus (ZIKV) infection during first trimester of pregnancy triggers disparate cellular response in placental disc CD45^+^ cells. **(a)** Relative proportions of immune subsets in placental disc CD45^+^ cells in ZIKV‐infected patients (*n* = 3) and healthy controls (*n* = 2). **(b)** Principal component analysis (PCA) of gene expression profiles of placental disc CD45^+^ cells from ZIKV‐infected patients and healthy controls. The PCA was performed on 30 717 genes selected by standard deviation > 0. **(c)** Correlation plot of immune‐cell subset frequencies in placental disc CD45^+^ cells with the first two principal components (PC1 and PC2) of the gene expression profiles. **(d)** Enrichment plot of the gene set GSE2405_0H_VS_9H_A_PHAGOCYTOPHILUM_STIM_NEUTROPHIL_DN, which was reported by GSEA as most enriched among all immunologic gene sets (C7 of MSigDB) in the placental disc CD45^+^ fraction of Patient 1 vs. healthy placenta. The profile shows the running enrichment score (green curve) and the positions of gene set members (black vertical bars) on the rank‐ordered list of differential gene expression comparing the Patient 1 vs. healthy placental disc CD45^+^ fraction.

To determine the percentages of various immune populations, immunophenotyping of the isolated CD45^+^ cells was performed (Figure 5a and Supplementary figure 5). Correlating these immunophenotyping data with the first two principal components of the gene expression data (Figure 5b) corroborated that the increase in neutrophil numbers (Figure 5c) was clearly associated with gene expression changes observed in the placenta from Patient 1 compared to healthy placentas. In the foetal membrane of the placenta, several transcripts encoded by inflammatory response genes and pathways were increased in abundance, including NFKB1, IL‐1B, TNF, TLR2, TLR4, CXCL8, NLRP3 and NLRP6 (Supplementary figure 4b and Supplementary table 3). Taken together, these data suggest that ZIKV infection during the first trimester of pregnancy could have led to an upregulated abundance of transcripts targeting eIF2 signalling, oxidative phosphorylation and neutrophil numbers. These findings would need to be corroborated with larger sample sizes in the future.

## Discussion

This study recruited three pregnant women who were infected with ZIKV during different trimesters of pregnancy. Despite the low number of patients recruited (one patient per trimester), this remains an excellent opportunity to obtain an insight into the impact of ZIKV infection during different trimesters on host cellular responses. Furthermore, the infection was caused by a phylogenetically different ZIKV strain compared to those reported in the Americas.^19^ Nevertheless, a series of histologic, immunologic and transcriptomic analyses were used to determine the impact of ZIKV infection on pregnancy progression and the cellular responses of the host towards the infection. All three babies were born at term with no observable birth defects, and all of the ZIKV‐infected mothers were clinically well, as with majority of ZIKV‐infected patients.^20^ However, different degrees of infection were apparent in the level of CD14^+^ monocytes between the three patients. This difference led to a disparate immune response exhibited by the patients. As confirmed by MFA of immune‐phenotyping and cytokine data, the three pregnant patients can be easily separated based on their immune response. Importantly, by comparing their cytokine profiles to those of the healthy non‐pregnant female controls, several immune mediators such as IL‐1RA, EGF, RANTES and IP‐10 were associated with the acute phase of ZIKV infection, a finding that was also observed in ZIKV‐infected non‐pregnant patients.^20^ Interestingly, EGF and IP‐10 are typically expressed at higher levels in women carrying a foetus with developmental anomalies.^21^ The association of neutrophils with the acute phase should also be given more attention since most studies have focused on the role of monocytes during ZIKV infection thus far.^22^, ^23^ Neutrophils have been shown to infiltrate the central nervous system of ZIKV‐infected immunocompromised mice^24^ and may therefore be a causative element in Zika‐induced neurologic damage.

Zika virus NS3 antigen was detected in placental tissues of all three patients at full term, confirming a strong tropism of the virus to the placenta regardless of the trimester of infection.^25^ The ZIKV NS3 antigen was detected in Hofbauer cells, placental macrophages,^9^ but not in trophoblasts. The susceptibility of trophoblastic cells to ZIKV infection is still uncertain, with reports indicating either permissive or resistance towards ZIKV infection.^9^, ^26^, ^27^ Interestingly, the localisation of the ZIKV NS3 antigen to CD163^+^ Hofbauer cells did not agree with the positive detection of ZIKV RNA load in the placental tissue. This finding may indicate that the virus has been cleared and the NS3 antigens are merely phagocytosed, non‐infectious viral proteins. This observation was further supported by the lack of any pathological insults (villitis, intervillositis, chorioamnionitis or funisitis) to the placental tissues in any of the three patients and the lack of detectable viral RNA by RNA‐seq, performed at the resolution sequenced. This observation also indicated an absence of overt inflammation, which permitted the pregnancy to progress to term without complications. This result was further confirmed by the absence of Hofbauer cell‐mediated hyperplasia in the chorionic villi, which has been previously associated with progression of congenital ZIKV infection.^27^

The transcriptomic profiling of placental cells showed that the cellular responses in Patient 1 (infected during the first trimester) were quite different from the other two patients and healthy controls for all three cell types analysed: placental disc CD45^+^, placental disc CD45^−^ and foetal membrane cells. GSEA revealed that a subset of genes in the CD45^+^ cells of Patient 1 (Figure 5d) resembled those documented in the bacterium *Anaplasma phagocytophilum*‐stimulated human primary neutrophils.^28^ This finding is not surprising, given that isolated CD45^+^ cells comprise a high proportion of neutrophils and thus would contribute the most to the transcriptome. More importantly, this finding also indicates the vast number of neutrophils present in the placenta during pregnancy. Their participation should not be taken lightly as this cell type may be involved in infertility, preeclampsia and even foetal loss. Neutrophils also participate in implantation, angiogenesis, spiral artery modification and parturition.^29^

The DEGs in the placental disc, both CD45^+^ and CD45^−^ fractions, and foetal membrane of Patient 1 were strongly associated with eIF2 signalling, oxidative phosphorylation and mitochondrial dysfunction. eIF2 signalling is crucial in regulating translational initiation leading to protein synthesis, and participates in cellular stress responses, erythropoiesis and immune responses to viral infection.^30^ Perturbed eIF2 signalling in placental development has been associated with intrauterine growth restriction of developing foetuses.^31^ In rats, eIF2 signalling was also shown to be heavily involved in the regulation of placental and foetal development.^32^ Likewise, oxidative phosphorylation and mitochondrial dysfunction have been associated with a range of gestational disorders.^33^ Mitochondrial dysfunction could also indicate an impairment of the antiviral immune response.^34^ It is known that some viruses attack the host cell mitochondrial network as a form of immune evasion.^35^, ^36^

The findings described herein can be translated into the clinical prenatal screening procedures in ZIKV‐infected or any arbovirus‐infected pregnant patients. In such circumstances, abnormalities in the numbers of leucocytes^37^, ^38^ and biomarkers indicative of ongoing inflammation^39^ or oxidative stress^40^ and presence of any microbial infections^41^ should be screened as advised by the clinic. Likewise, RNA from the amniotic fluid or amniocytes can be profiled to determine the transcript abundance of molecules involved in important biological pathways.^42^ Adopting these steps will potentially improve the clinical care administered to affected patients, by identifying potential danger signs associated with ZIKV infection.

## Methods

### Ethics approval and consent to participate

Written informed consent was obtained from all participants in accordance with the Declaration of Helsinki for human research. The study protocol was approved by the SingHealth Centralized Institutional Review Board (CIRB Ref: 2016/2219). Blood samples were collected from healthy donors who had provided written consent, in accordance with the guidelines of the Health Sciences Authority of Singapore (study approval number: NUS IRB: 10‐250).

### Patients and sample collection

Three pregnant women were enrolled into the study upon admission to the KK Women's and Children's Hospital, Singapore. Patient 1 was 6‐weeks pregnant, Patient 2 was 17‐weeks pregnant, and Patient 3 was 35‐weeks pregnant when they presented symptoms of ZIKV infection. Upon admission, patients were confirmed to be infected via qRT‐PCR detection of ZIKV RNA in their urine. Whole blood samples were collected at two time points: 0–7 days PIO and 10–14 days PIO, representing the acute and convalescent phases, respectively. Whole blood was collected in EDTA Vacutainer tubes (Becton Dickinson, Franklin Lakes, NJ, USA) after peripheral venipuncture. Each sample was first used for blood count, whole blood staining and viral load quantification and then centrifuged at 256 **g** for 10 min to collect plasma for storage at −80°C. Whole blood samples were also collected from three healthy women and pre‐screened for ZIKV viral RNA and ZIKV‐specific antibodies using in‐house protocols.^13^ Placentas from two healthy individuals were obtained as control. All healthy donors were non‐febrile and had no signs of acute illness during recruitment.

### Placenta processing

After successful full‐term delivery, the placenta was maintained on ice and transported to the Singapore Immunology Network for immediate downstream processing within 2 h of expulsion. The term placenta was first separated into the foetal membrane (chorion and amnion) and placental disc (chorionic villi) layers. A small section of the tissue was removed and placed in 10% neutral buffered formalin (NBF; Sigma‐Aldrich, Saint Louis, MO, USA) for histology and immunofluorescence staining. The remaining tissues were finely diced in a digestion medium containing collagenase type 4 (0.2 mg mL^−1^) and DNase (0.05 mg mL^−1^) prepared in Roswell Park Memorial Institute medium (GE Healthcare Life Sciences, Pittsburgh, PA, USA) containing 10% foetal bovine serum (Thermo Fisher Scientific, Waltham, MA, USA). Refer to [Link], [Link], [Link], [Link], [Link], [Link], [Link], [Link] for more information.

The foetal membranes were incubated in digestion medium for 3 h in a 37°C incubator supplemented with 5% CO_2_. The foetal cells were then centrifuged (400 *g*, 5 min) and repeatedly washed with PBS to remove traces of digestion medium before red blood cell (RBC) lysis. Lysis was done with in‐house RBC lysing solution containing 155 mm NH_4_C1 (Sigma‐Aldrich), 10 mm KHCO_3_ (Sigma‐Aldrich) and 0.1 mm EDTA (Thermo Fisher Scientific). Lysed cells were then enumerated and used for downstream procedures.

The placental disc was incubated in digestion medium for 1 h, and then, the digestion medium was carefully overlaid on Ficoll‐Paque Plus (GE Healthcare Life Sciences) at a ratio of 2:1, respectively. The samples were then centrifuged at 1600 *g* for 20 min (with minimal acceleration and deceleration) to isolate the ‘buffy coat’ containing leucocytes. The leucocytes were carefully removed, and traces of RBCs were subsequently lysed. The cells were enumerated and used for downstream procedures.

In both cases, the digestion medium was filtered through a 100‐μm filter unit after digestion to remove any undigested tissues. The volume of medium used was adjusted according to the size of the tissue being digested.

### Histology and immunofluorescence

Placental tissues were first fixed in 10% NBF (Sigma‐Aldrich) at room temperature for 24 h before being processed for routine histologic evaluation. Briefly, isolated tissues were embedded in paraffin wax, cut into 5‐μm‐thick sections, deparaffinised and then stained with H&E. The stained sections were viewed under an Olympus BX53 upright microscope (Olympus Life Science, Tokyo, Japan), and images were captured with an Olympus DP71 digital camera using an Olympus DP controller and DP manager software. All images were evaluated by a certified pathologist.

Tissue sections were stained by standard immunofluorescence technique. In brief, antigen retrieval was performed after deparaffinisation, using DAKO target retrieval solution, pH9. After washing with TBS‐T, the tissues were then treated with 10% goat serum for 30 min to block the non‐specific reaction. The tissues were then incubated overnight with various combinations of a ZIKV in‐house antibody^13^ (1:100) and CD163 (Thermo Fisher Scientific; 1:50) at 4°C. The slides were incubated with secondary antibodies (1:500) such as Alexa Fluor^®^ 488 goat anti‐rabbit IgG (Thermo Fisher Scientific) and Alexa Fluor^®^ 594 goat anti‐mouse IgG (Thermo Fisher Scientific) for 30 min in the dark, and the nuclei were counter‐stained with Vectashield™ Hard Set mounting medium with DAPI. The stained slides were examined under Nikon Eclipse 90i fluorescence microscope (Nikon, Tokyo, Japan), and images were captured with microscopic camera, DS‐Fi3 (Nikon), using NIS‐Elements imaging software (Nikon).

### Blood count

A complete blood count (CBC) was determined using an Ac·T diff haematology analyser (Beckman Coulter, California, CA, USA), according to the manufacturer's instructions. Beckman Coulter 4C© Plus Tri‐Pack Cell Controls (Beckman Coulter) were used to confirm instrument accuracy and precision.

### Whole blood labelling and flow cytometry

Whole blood (100 μL) was labelled as previously described.^13^ Antibodies were used to identify CD45^+^ leucocytes (mouse anti‐human CD45; Biolegend, California, CA, USA), high SSC‐A CD16^+^ neutrophils (mouse anti‐human CD16; Biolegend), CD14^+^ monocytes (mouse anti‐human CD14; BD Biosciences, New Jersey, NJ, USA), CD3^+^ T cells (mouse anti‐human CD3; BD Biosciences), CD4^+^ T helper cells (mouse anti‐human CD4; Thermo Fisher Scientific), CD56^+^ NK cells (mouse anti‐human CD56; Miltenyi Biotec, Bergisch Gladbach, Germany) and CD19^+^ B cells (mouse anti‐human CD19; Thermo Fisher Scientific). Cell fixation and RBC lysis was performed using 1× FACS lysing solution (BD Biosciences), and permeabilisation using 1× FACS permeabilisation solution 2 (BD Biosciences) before staining with a ZIKV NS3 protein‐specific rabbit polyclonal antibody.^13^ The labelled cells were counter‐stained with a fluorophore‐tagged secondary goat anti‐rabbit IgG (H + L) antibody (Thermo Fisher Scientific), before acquisition on a LSR Fortessa (BD Biosciences). Dead cells were excluded using a Live/Dead™ Fixable Aqua Dead Cell Stain Kit (Thermo Fisher Scientific). The frequencies of peripheral blood immune subsets were obtained with the following formula: Percentage of specific immune subset (obtained from immune‐phenotyping) × total leucocyte number (obtained from CBC) = cellular number of specific immune subset.

### Multiplex microbead immunoassay for cytokine quantification

Cytokine and chemokine levels in plasma from ZIKV‐infected patients were measured simultaneously using a multiplex microbead‐based immunoassay, ProcartaPlex Human Cytokine/Chemokine/Growth Factor Panel 1 (Thermo Fisher Scientific), as previously described.^20^ Plasma samples and reagents were prepared, and immunoassay procedures were performed according to the manufacturer's instructions.

### ZIKV virion‐based ELISA

The presence and titres of ZIKV‐specific antibodies in the plasma of the infected pregnant women at both acute and convalescent phases were determined using a ZIKV virion‐based ELISA, as previously described.^13^ Plasma samples were tested at 1:200 dilutions.

### RNA sequencing (RNA‐seq)

RNA samples, extracted from the fractions of isolated placental cells, were treated with DNase using an Ambion Turbo DNA‐free Kit (Thermo Fisher Scientific) and then purified using Ampure XP beads (Beckman Coulter). The DNase‐treated RNA (2 μg) was treated with Ribo‐Zero using an Epicentre Ribo‐Zero Gold Kit (Human/Rat/Mouse; Illumina, San Diego, CA, USA) and re‐purified on Ampure XP beads. Successful RNA depletion was verified using a Qubit (Thermo Fisher Scientific) and an Agilent 2100 Bioanalyzer (Agilent, Santa Clara, CA, USA), and all depleted RNA was used as input material for the ScriptSeq v2 RNA‐Seq Library Preparation protocol. RNA was amplified for 14 cycles, and the libraries were purified on Ampure XP beads. Each library was quantified using a Qubit, and the size distribution was assessed using an AATI Fragment Analyser (Agilent); the final libraries were pooled in equimolar amounts. The quantity and quality of each pool was assessed using a Fragment Analyser and by qPCR using an Illumina Library Quantification Kit (KAPA Biosystems, Wilmington, Massachusetts, USA) on a LightCycler LC480II (Roche, Basel, Switzerland), according to the manufacturer's instructions. The template DNA was denatured according to the protocol described in the Illumina cBot User guide and loaded at a 12 pm concentration. Sequencing was carried out on three lanes of an Illumina HiSeq 2500 with version 4 chemistry, generating 2 × 125 bp paired‐end reads.

### RNA‐seq data analysis

Quality assessment of the RNA sequencing reads was performed using FASTQC (version 0.11.5; https://www.bioinformatics.babraham.ac.uk/projects/fastqc/). Cutadapt (version 1.2.1)^43^ was used to remove Illumina adapter sequences with option ‐O 3 so that the 3′ end of any reads that matched the adapter sequence for ≥ 3 bp was trimmed. The reads were further trimmed using Sickle (version 1.20; https://github.com/najoshi/sickle) with a minimum window quality score of 20. Reads < 10 bp after trimming were removed. The resulting clean, paired‐end reads were mapped to human transcript sequences obtained from Gencode (version 27)^44^ using Salmon (version 0.9.1).^45^ Transcript abundances quantified by Salmon were summarised to gene‐level counts and normalised gene‐level abundances in TPM units using the tximport R/Bioconductor package (version 1.2.0).^46^

Principal component analysis was performed on all samples using genes with TPM > 1 in at least one sample. The TPM values were log_2_‐transformed after adding a pseudocount of 1.0 to prevent negative values and used for PCA in FactoMiner R package (version 1.33)^47^ with default scaling transformation.

Differential gene expression analysis was performed using edgeR R/Bioconductor package (version 3.16.5).^48^ Gene‐level counts for samples relevant to the conditions being compared were loaded into edgeR, and a negative binomial (NB) generalised linear model extended with quasi‐likelihood (QL) methods was fitted to the count data. As recommended for gene‐level counts summarised using tximport, the transcript abundances and average transcript lengths of genes were used to specify the offset term in the edgeR model in order to correct for potential differences in transcript usage between samples.^46^ Low expressed genes with < 5 counts in all samples were removed. The trended NB dispersion was estimated using the estimateDisp function, and the QL dispersion was estimated using the glmQLFit function. DGE was determined using a QL *F*‐test (glmQLFTest function), which provides a conservative and rigorous type I error rate control.^48^ Genes with a Benjamini–Hochberg multiple testing corrected *P*‐value < 0.05 were considered differentially expressed. DEG lists were analysed with DAVID GO analysis (version 6.8),^49^ Ingenuity Pathway Analysis^®^ and GSEA (version 3.0)^50^ to identify associated biological conditions and pathways. The curated gene sets used for GSEA included the hallmark (H) and immunologic signature (C7) gene sets downloaded from Broad Institute's Molecular Signatures Database that accompanies the GSEA software.

### Multiple factor analysis

Data from immunophenotyping of whole blood and cytokine quantification in plasma were analysed together using MFA^16^; MFA permits several groups of variables to be studied on the same set of individuals. Analysis was performed with nine individual samples, including the three ZIKV‐infected patients in the acute and convalescent phases of infection and the three healthy controls. The immunophenotyping and cytokine measurements were specified as two different groups of variables consisting of log_10_‐transformed cell counts per μL of whole blood and log_10_‐transformed cytokine levels in pg mL^−1^ units. The analysis was carried out using R package FactoMineR (version 1.33)^47^ with the default parameters. The data were plotted using custom R scripts. Data from placental immunophenotyping and RNA sequencing were also analysed using MFA. Data acquired from placental disc and foetal membrane fractions were analysed separately. The individuals in this analysis included the three ZIKV‐infected patients and two healthy controls. Cellular fractions were expressed as a percentage of the parent gate in FACS analysis, and RNA‐seq measurements were expressed in log_2_ TPM units.

## Conflict of interest

The authors declare no conflict of interest.
