# Supplementary figures and images for: Immunological observations and transcriptomic analysis of trimester‐specific full‐term placentas from three Zika virus‐infected women
Source: Clin Transl Immunology. 2019 Nov 5;8(11):e01082. doi: 10.1002/cti2.1082 (PMC6831931; doi:10.1002/cti2.1082)

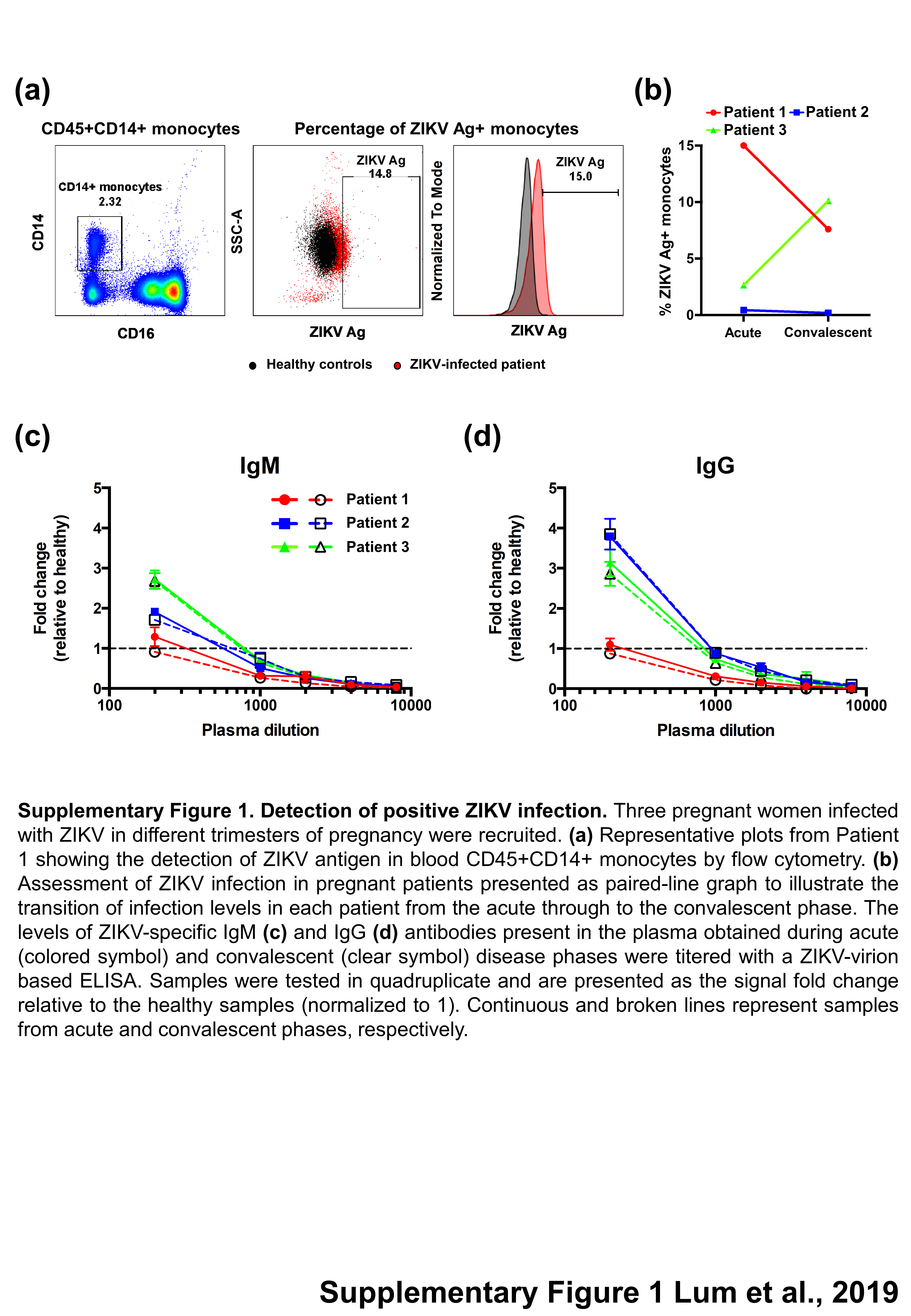

Supplement: Supplementary file 1 [file CTI2-8-e01082-s001.tif]

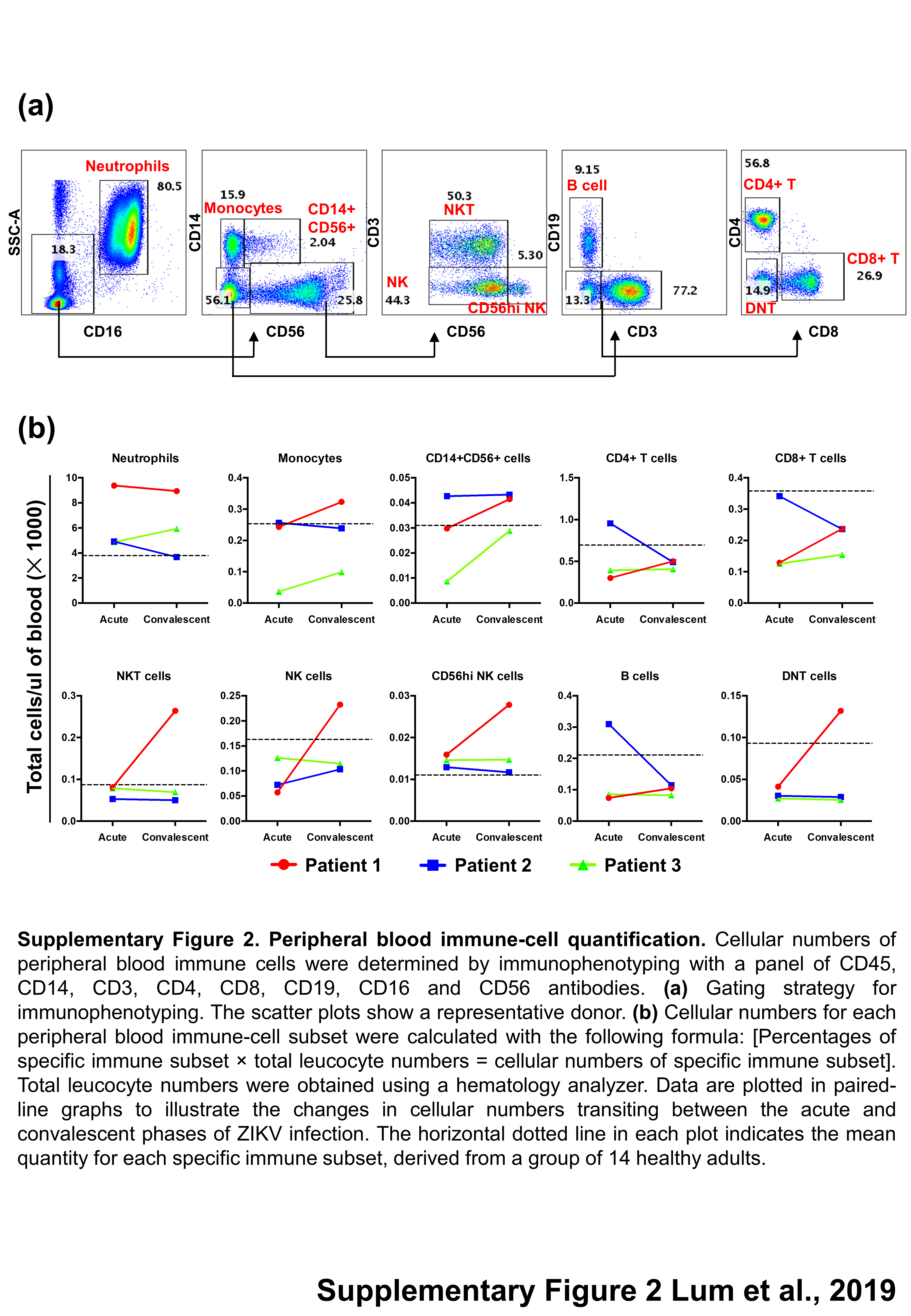

Supplement: Supplementary file 2 [file CTI2-8-e01082-s002.tif]

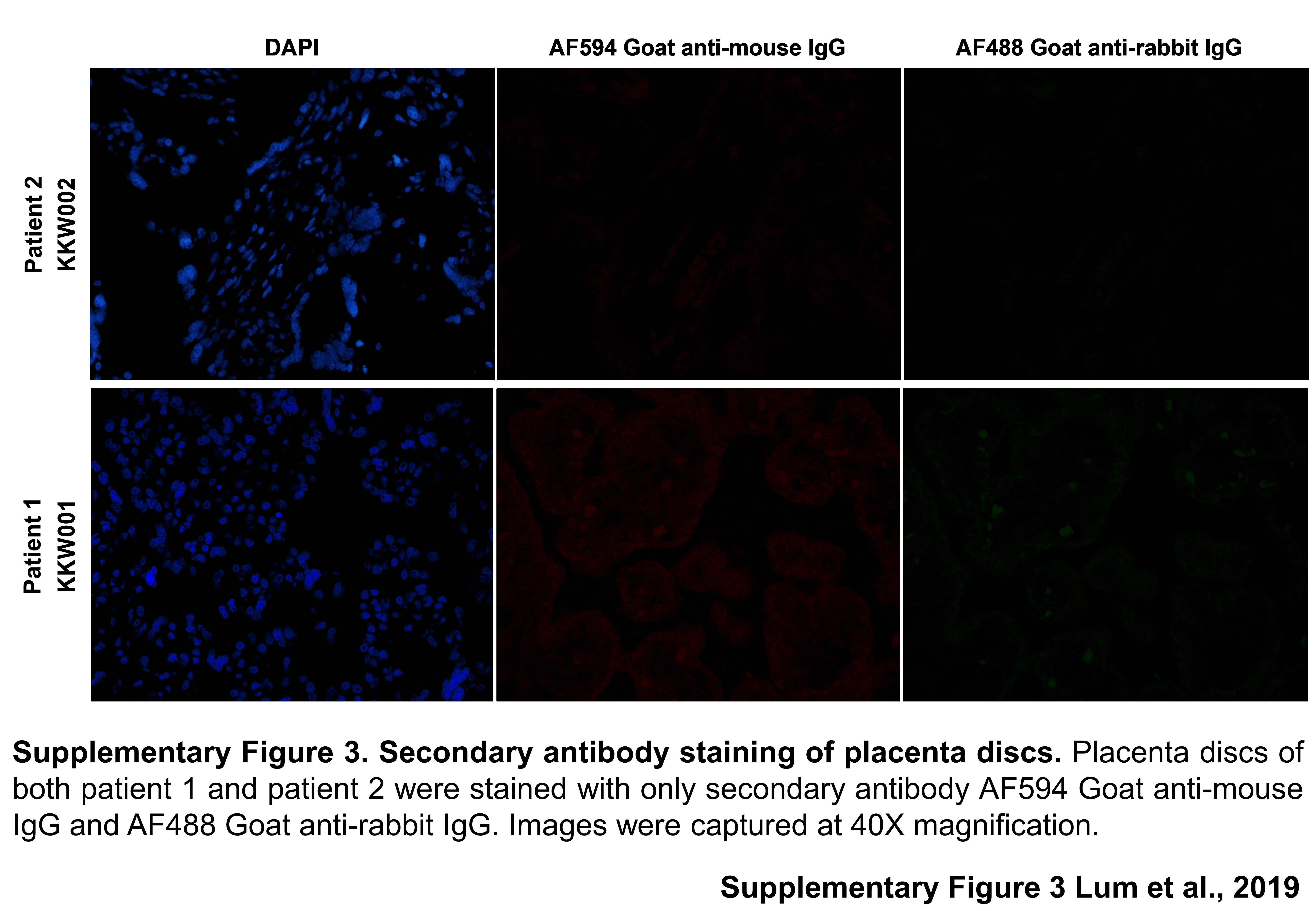

Supplement: Supplementary file 3 [file CTI2-8-e01082-s003.tif]

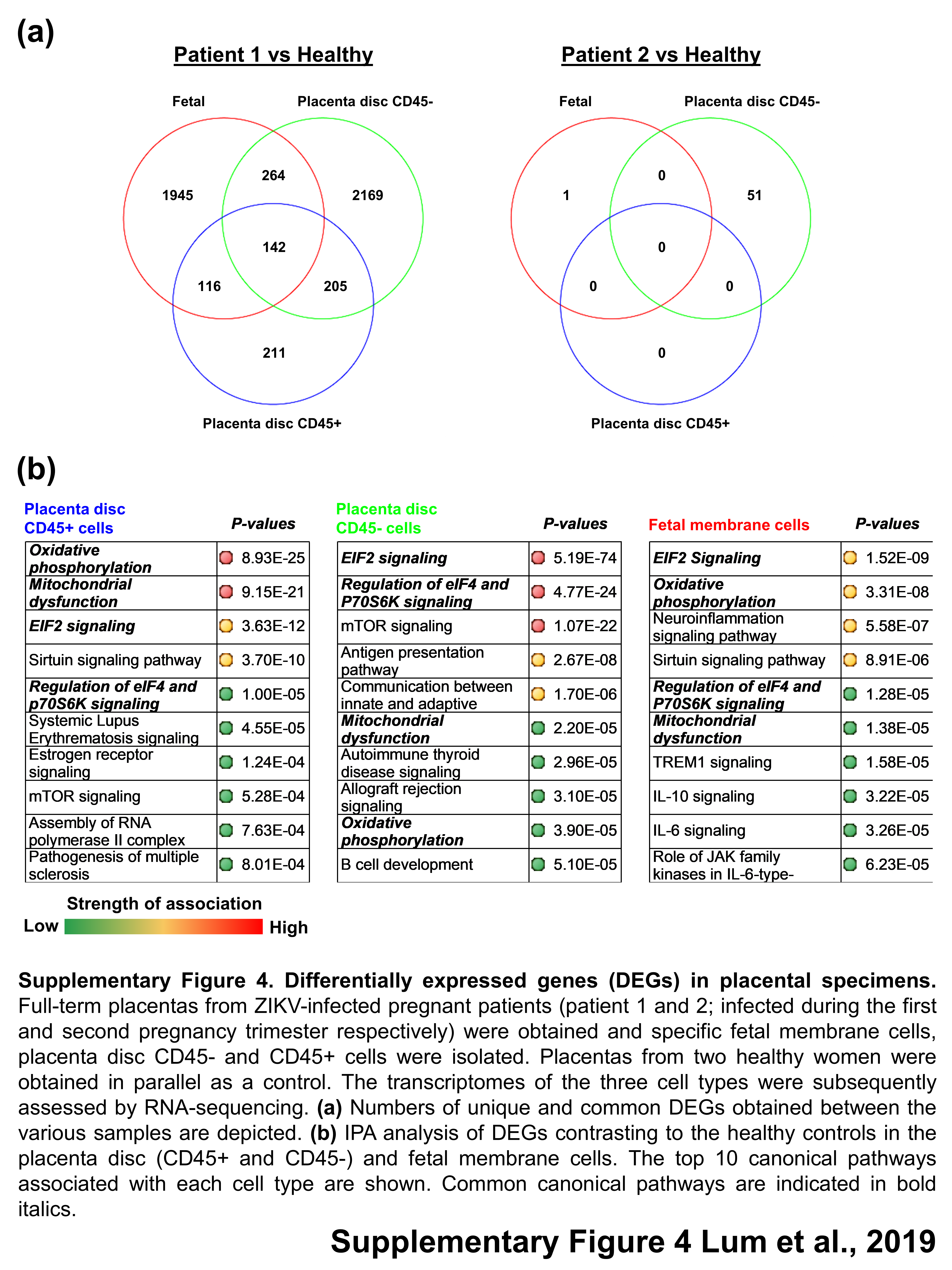

Supplement: Supplementary file 4 [file CTI2-8-e01082-s004.tif]

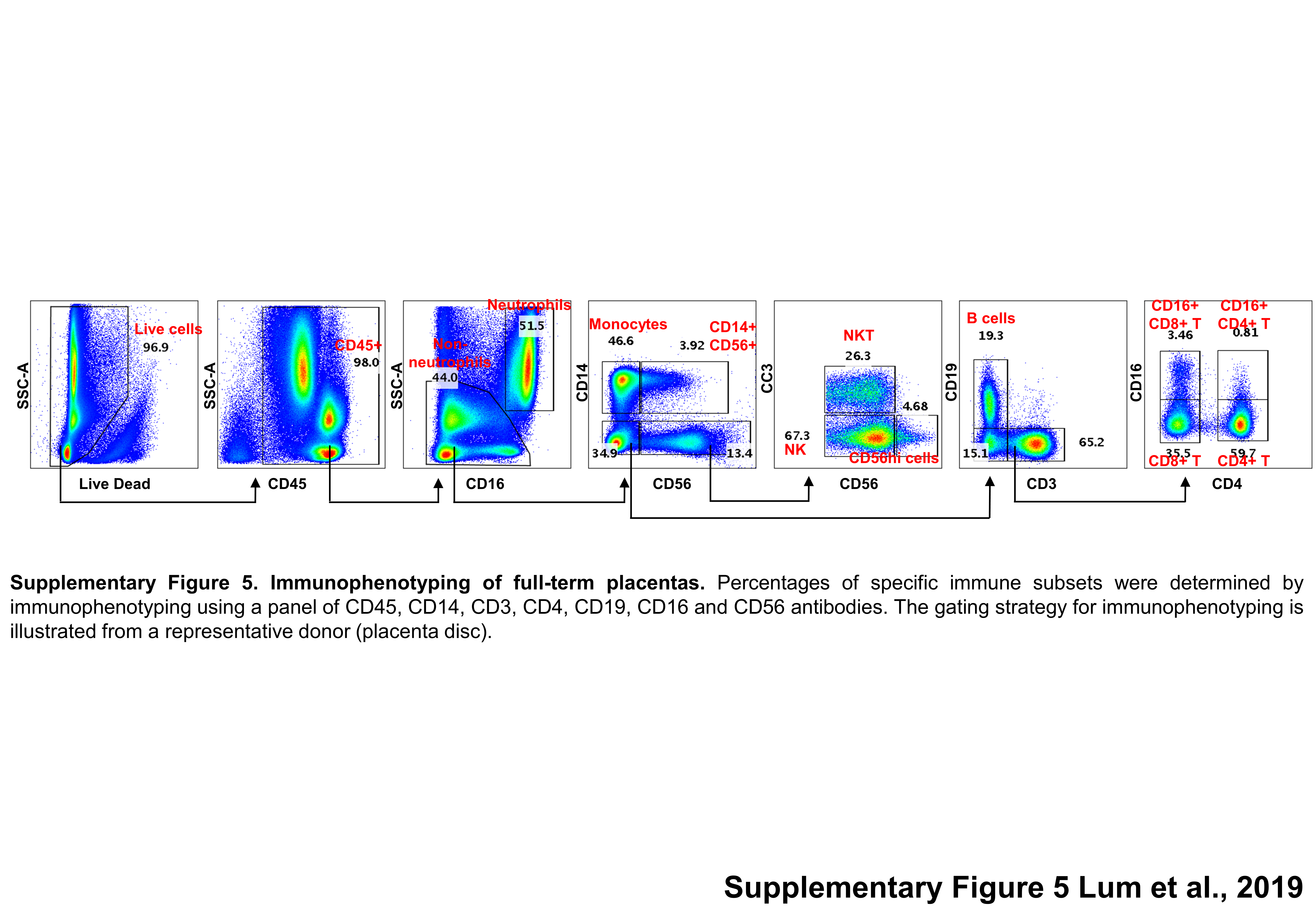

Supplement: Supplementary file 5 [file CTI2-8-e01082-s005.tif]
